# Impact of COVID-19 pandemic on bone marrow transplantation in Morocco

**DOI:** 10.11604/pamj.2020.35.5.22619

**Published:** 2020-04-16

**Authors:** Maryame Ahnach, Kamal Doghmi

**Affiliations:** 1 Department of Hematology, International University Hospital Cheikh Khalifa Ibn Zayd, Casablanca, Morocco; 2 Mohammed VI University of Health Sciences, Casablanca, Morocco

**Keywords:** COVID 19, allogenic, autologous, stem cell transplantation

## To the editors of Pan African Medical Journal

The novel coronavirus (COVID-19) pandemic is the defining global health crisis of our time and the greatest threat we have faced during this century. As a highly contagious virus, the infection emerged in China in January 2020 [1] and rapidly spread globally; with the most affected regions being the USA, Europe, Republic of Korea and Iran. According to the World Health Organizations´ (WHO) data, in April 2020, one million of the population has been infected with more than 50 000 deaths [2]. To date, in the absence of any specific treatment, our knowledge about this disease remains very limited and is subject to rapid change. Cancer is considered a risk factor for COVID-19 infection (1%) [3], with very few cases reported in hematology-Oncology, but with no data related to bone marrow transplant. This virus represents a serious danger for patients with hematologic malignancies scheduled for bone marrow transplant due to myeloablative conditioning and immunosuppressive treatments. People receiving chemotherapy with compromised immune systems and complications after stem cell transplant have an increased risk for infection [4]. During a bone marrow transplant, pulmonary complications are frequent and associated to death [5]. COVID-19 infection may complicate clinical symptoms with higher risk of respiratory distress [6], and this situation could be even more critical depending on factors of co-morbidity such as age, cardiovascular, liver and kidney diseases [7]. In addition to the virulence of the infection, restrictive government measures cause many obstacles and difficulties for the transplant course: 1) blood transfusion is vital for transplant patients; the number of blood donors decreases drastically with population confinement, 2) drug manufacturing and available medicines are compromised, 3) the allogenic stem cell transplant with donors on international files becomes very difficult to access because of the international borders shut down, 4) Management of unstable patients requires an intensive care unit with mechanical ventilation, however, depending on the pandemic level and the healthcare system in each country, hospital beds might be lacking.

Therefore, the management of patients transplanted during a pandemic is very complex. To overcome this crisis, some recommendations and emergency measures have been developed by scientific societies like the European Society for Blood and Bone Marrow Transplantation [8,9]. Their guidelines are established according to the coronavirus high contagion rate in Europe. The most important messages are the postponement of any low-risk non-urgent transplant, and freezing rich grafts if mobilization is already scheduled. Currently, virus detection has become mandatory before any transplant process, among donors as well as recipients for allogenic stem cell transplant using throat-swab specimens for PCR test. During the transplant period, detection must be carried out before any symptom like fever, cough or chest imaging abnormalities. Prevention procedures remain a very useful mean to avoid infection, and the WHO recommendations must be diligently followed by healthcare staff, patients and donors. It is vital to be very careful with hygiene routines, including hand washing, use of protective mask, alcohol-containing hand sanitizers, and limited visits. In post-transplant follow-up period, it is recommended to prioritize telemedicine consultations if possible. In Morocco, the situation is less critical than in Europe, with a record of 1184 confirmed cases with 90 deaths [10]. The government has adopted containment measures very early but the contagion is still gaining ground, and to ensure the safety of our transplanted patients, our hematology centers have adopted actions based on the European recommendations. In the Table 1, we summarize the main practical steps that can be taken to reduce the risk to our vulnerable patients during bone marrow transplant [8].

**Table 1:** practical recommendations for bone marrow transplant during COVID-19 pandemic

**Prioritizing stem cell transplant**	- Prioritize urgent patients with high risk of disease progression and high grade malignancy.
	- Defer transplant if possible, for patients with low risk progression, poor outcome or high risk of immunosuppression.
	- Make decisions and discuss the risks and benefits as part of a multidisciplinary team.
**Hygiene Procedures**	- Follow strict hygiene procedures for patients, donors, medical staff and visitors.
	- Isolate suspected patients and infected persons.
	- Limit access to unit transplants.
**Recommendations for recipients**	- COVID-19 testing in all candidates before any steps of transplantation.
	- Defer transplant in positive patients, and in any suspect contact or contagion of COVID-19.
	- Test and explore any patients with symptoms of COVID-19.
**Recommendations for donors**	- Exclude positive person and suspected contagion.
	- Test all donors before harvesting stem cell.
	- Mobilization of stem cell with growth factors without chemotherapy.
	- Collect a rich graft and cryopreservation if possible.
**Treatment of COVID-19 Infection**	- No guidelines for treatment.
	- Potential treatment with hydroxychloroquine with azithromycin, lopinavir/ritonavir, tocilizumab.

## Conclusion

The situation is critical all over the world and restrictive measures will affect bone marrow transplantation as the COVID-19 is spreading. It is necessary to carefully follow all international recommendations; Morocco has therefore taken emergency measures to minimize the impact of COVID-19 on transplant activity.

## Competing interests

All authors declare no competing interests.
